# A GENDERED PERSPECTIVE ON INTERGENERATIONAL RELATIONSHIPS IN THE SECOND HALF OF LIFE

**DOI:** 10.1093/geroni/igad104.0253

**Published:** 2023-12-21

**Authors:** Liat Ayalon

**Affiliations:** Bar Ilan University, Ramat Gan, HaMerkaz, Israel

## Abstract

Life satisfaction is defined as the perception of individuals concerning the level of meaning, fulfillment, and satisfaction in their life. It has shown to be an important indicator of older persons’ perceived ability to pursue their life goals. The present study evaluated the relationship between older mothers’ and fathers’ perceptions of their relationship with their children and their life satisfaction four years afterwards. We relied on the Health and Retirement Study, which is a representative sample of US citizens at the age of 50 and over. In total 1071 continuously married couples participated in both 2006 and 2010 waves and completed the leave behind questionnaire. Relying on path analysis, our findings show that fathers’ life satisfaction in 2010 is associated with perceived support from their children in 2006. Mothers’ life satisfaction in 2010 on the other hand, is associated with perceived support from their children as well as with lower levels of perceived strain in the relationship with their children in 2006. Results are discussed in relation to gender roles in the second half of life as manifested in intergenerational relationships.

